# Species and Media Effects on Soil Carbon Dynamics in the Landscape

**DOI:** 10.1038/srep25210

**Published:** 2016-05-03

**Authors:** S. Christopher Marble, Stephen A. Prior, G. Brett Runion, H. Allen Torbert, Charles H. Gilliam, Glenn B. Fain, Jeff L. Sibley, Patricia R. Knight

**Affiliations:** 1 University of Florida/IFAS, Mid-Florida Research and Education Center, FL 32703, Apopka, USA; 2 USDA-ARS National Soil Dynamics Laboratory, AL 36832, Auburn, USA; 3 Auburn University Dept. of Horticulture, AL 36849, Auburn, USA; 4 Mississippi State Coastal Research and Extension Center, MS 39532, Biloxi, USA

## Abstract

Three woody shrub species [cleyera (*Ternstroemia gymnanthera* Thunb. ‘Conthery’), Indian hawthorn (*Rhaphiolepis indica* L.) and loropetalum (*Loropetalum chinensis* Oliv.‘Ruby’)] were container-grown for one growing season in 2008 using either pinebark (industry standard), clean chip residual or WholeTree (derived by-products from the forestry industry) as potting substrates and then transplanted into the landscape in 2008. An Automated Carbon Efflux System was used to continually monitor soil CO_2_ efflux from December 2010 through November 2011 in each species and substrate combination. Changes in soil carbon (C) levels as a result of potting substrate were assessed through soil sampling in 2009 and 2011 and plant biomass was determined at study conclusion. Results showed that soil CO_2_-C efflux was similar among all species and substrates, with few main effects of species or substrate observed throughout the study. Soil analysis showed that plots with pinebark contained higher levels of soil C in both 2009 and 2011, suggesting that pinebark decomposes slower than clean chip residual or WholeTree and consequently has greater C storage potential than the two alternative substrates. Results showed a net C gain for all species and substrate combinations; however, plants grown in pinebark had greater C sequestration potential.

There is growing concern that anthropogenic driven changes in the earth’s surface temperatures may impact the future global environment^1^,^2^. Agricultural lands occupy 37% of the earth’s land surface and significantly contribute to greenhouse gas (GHG) emissions of carbon dioxide (CO_2_), methane (CH_4_) and nitrous oxide (N_2_O)^3^,^4^,^5^. To help mitigate climate change, emissions from agriculture must be reduced along with increased long-term C capture and storage (sequestration)^6^,^7^. In contrast to other industries, agriculture is unique in that activities which were once net CO_2_ sources can potentially become net sinks by employing management practices^8^ that lead to long-term C storage in biomass, wood products, soils and forests^9^.

Extensive research has focused on reducing GHG emissions and increasing carbon (C) storage in agricultural production^1^,^10^,^11^,^12^,^13^,^14^,^15^,^16^,^17^. Due to their large land area coverage in the U.S., most C sequestration research has focused on row crop (113 million ha) and forest (300 million ha) production systems^18^,^19^. However, non-agricultural U.S. land (e.g., urban and suburban) comprises approximately 60 million ha^20^. As such, a significant proportion of this land is (or could be) planted with ornamental trees and shrubs, but little research has investigated ornamentals in these settings.

Since many North American cities expand at twice the rate of population growth, urban migration is causing rural land to be consumed by suburban areas^21^. Exponential growth in urban and suburban areas has led researchers to begin investigating the influence of these areas on environmental change. Human activity and CO_2_ emissions are usually highest in cities and industrial areas (~2% of the earth’s surface) and represent 30–40% of emitted anthropogenic GHGs^22^. Crawford *et al.*^21^ suggested that suburban areas, with their large land mass and populations, are an essential component of CO_2_ emission calculations. Previous research has identified urban forests as a significant C storage system through accumulation in growing tree biomass^23^,^24^,^25^. Nowak and Crane^26^ previously showed that urban trees in the U.S. stored ~700 million metric tons of C with an annual sequestration rate of 20.8 million metric tons of C. In addition to storing C, urban trees have also been shown to reduce air pollution^27^ and cool ambient air, consequently reducing energy consumption in some areas by reducing heating and cooling costs^28^. While the role of large tree species in C capture and storage has been previously identified, it is important to note that many urban and suburban landscapes are dominated with woody shrubs. Whittinghill *et al.*^29^ reported that landscape shrubs such as *Spiraea media* Schmidt. ‘Darsnorm’, *Weigela florida* Bunge. ‘Alexandria’ and *Buxus* × ‘Green Velvet’ typically provide greater C storage benefits than green roof species (e.g., *Sedum* spp.) which are highly touted and promoted for their C sequestration potential. While there is some indication that ornamental shrubs may impact C sequestration, little or no data currently exist for most landscape species.

Another often ignored key C sequestration pathway (other than plant biomass) is the substrates these plants were grown in during production. Woody landscape plants are predominately container-grown using a soilless potting substrate [i.e. pinebark (PB)] which has a high C content (~50%). These substrates are interred when the plants are transplanted into the landscape thereby instantly storing C in the soil system. Marble *et al.*^30^ estimated that planting an average 11.5 L nursery container filled with a PB potting substrate would instantly sequester ~1.6 kg of C from the substrate alone, with future C gains realized as plant biomass increases.

Decreased demand in domestic forestry production has caused PB supplies to occasionally become scarce in recent years^31^. Consequently, other potting substrates, such as WholeTree (WT)^32^,^33^,^34^ and clean chip residual (CCR)^35^,^36^, have recently been shown to be suitable growth substrates for a wide variety of ornamental species. WholeTree and CCR have a C concentration similar to PB (~50% C)^37^ and would likely result in similar initial C gains following transplanting into the landscape. If C sequestration in agriculture is necessary to mitigate climate change, it is important to examine the contributions from all agricultural sectors, including specialty crop industries (e.g., ornamental horticulture) that influence many suburban areas. The objective of this study was to examine species and media effects on plant growth and soil C dynamics following transplanting of container-grown woody ornamental shrubs into the landscape. The null hypothesis tested was that neither species nor media would affect plant growth or soil C dynamics following transplanting of container-grown woody ornamental shrubs into the landscape.

## Materials and Methods

Three species of woody ornamentals [cleyera (*Ternstroemia gymnanthera* Thunb. ‘Conthery’), Indian hawthorn (*Rhaphiolepis indica* L.) and loropetalum (*Loropetalum chinensis* Oliv.‘Ruby’)] were transplanted from 7.6 cm liners into #1 (3.8 L) containers on April 4, 2008. Plants were containerized using one of three different growth substrates (PB, CCR, or WT). The C concentration of PB, CCR and WT was determined to be 49.2, 46.9 and 47.8%, respectively. The source, age, handling and processing of these substrates prior to potting was previously reported^38^.

**Figure 1:**
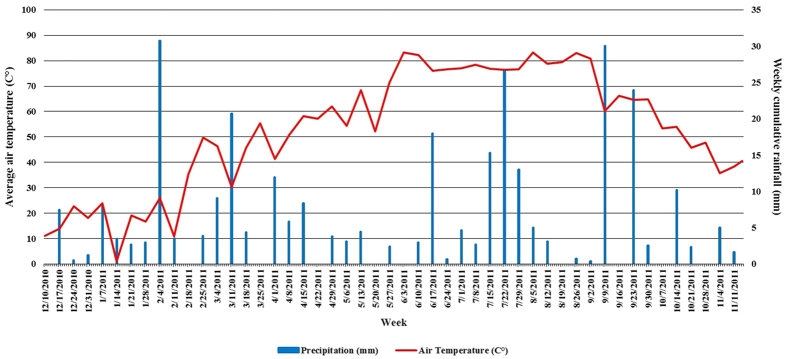
Average weekly air temperature (C°) and cumulative rainfall (mm).

On the day of potting, each substrate was mixed with sand on a 6:1 (v:v) basis and pre-plant incorporated with an 8 to 9 month formulation fertilizer [Polyon^®^ (18-6-12), Harrell’s Fertilizer Inc. Sylacauga, AL] at 18.3 kg·m^−3^, 3.0 kg·m^−3^ dolomitic limestone and 0.9 kg·m^−3^ Micromax^®^ micronutrient blend (Everiss International B.V., Geldermalsen, The Netherlands). Following transplanting, plants were placed on an outdoor gravel container pad and overhead irrigated twice daily (1.27 cm d^−1^). Plants were arranged by species in a randomized complete block design with 20 single pot replications per treatment and grown for nine months. In December 2008, six cleyera and loropetalum and eight Indian hawthorn plants from each substrate treatment were selected for field transplant; plants with a similar growth index [(plant height + plant width1 + plant width2)/3] were selected using Tukey’s Mean Separation Test (*P* < 0.05) (SAS^®^ Institute version 9.1, Cary, NC) (data not shown). Plants were transplanted by species into a clay-loam soil with a pH of 6.2 and 2.9% soil C at the demonstration farm on the campus of Auburn University, Auburn, AL. Cleyera and loropetalum were planted into single separate rows 0.9 m apart; Indian hawthorns were also placed in a single row and spaced 0.6 m apart. Approximately 3.5 L of the initial substrates mentioned above were placed in the landscape during transplanting. Plants within each species were arranged in a randomized block design with pairs of plots for each substrate randomized within each of three blocks for cleyera and loropetalum and each of four blocks for Indian hawthorn. Plants were manually watered following transplanting and received only rainfall thereafter. All plants were mulched with pine straw at transplanting (5 cm depth) and again on June 30, 2010. Plants were fertilized on June 25, 2009 by broadcasting an 8 to 9 month formulation fertilizer [Polyon^®^ (13-13-13), Harrell’s Fertilizer Inc. Sylacauga, AL) at a rate of 454 g of product per 93 m^2^. Weed control was conducted manually and by directed applications of glyphosate (RoundUp™ Pro, Monsanto Co., St. Louis, MO) herbicide at a 2% spray solution as needed. Weekly cumulative rainfall and average air temperature across the study period were calculated from data collected at a nearby (0.33 km) weather station (Fig. 1).

Soil CO_2_ efflux was measured using the Automated Carbon Efflux System (ACES; US Patent 6,692,970), developed by the USDA Forest Service (Southern Research Station Laboratory, Research Triangle Park, NC)^39^. Details of the ACES system used in this study have been previously reported^40^. Briefly, ACES is a chamber-based, multi-port respiration measurement system, which uses open system, dynamic soil respiration chambers measuring 25 cm diameter (491 cm^2^) equipped with air and soil thermocouples (inserted to 5 cm depth). The chambers have pressure equilibration ports to eliminate differences in chamber pressure that may compromise the quality of the respiration measurement^41^. In November 2010, the ACES system was installed to continuously monitor (24 hr d^−1^) C lost via soil respiration. An ACES sampling chamber was placed directly adjacent to a plant grown in each substrate in three blocks for each of three species, resulting in three replicated sampling chambers for each species/substrate combination; one Indian hawthorn block was not monitored due to system constraints. Additionally, three sampling chambers were placed in native bare-soil (BS) plots (one chamber in one BS plot by each species). Natural precipitation was allowed to reach the soil within the soil chambers by rotating each soil chamber between two sampling points on either side of each species/substrate combination on a weekly basis. Litter on the soil surface was not removed from any sample point, but all sample points were kept free of live vegetation throughout the study. The ACES units were operated continuously from December 5, 2010 until November 14, 2011, with the exception of brief periods when monitoring was interrupted due to power outages or for routine maintenance.

Belowground soil C and N was first assessed in June 2009. For each species, one soil core (3.8 cm diameter × 60 cm depth) was collected from the root-zone of each treatment in all blocks according to previously described methods^42^. Soil cores were also collected in the same manner to determine the BS (no species or substrate) C and N from all blocks within each species. All cores were divided into 15 cm depth segments, sieved (2 mm), oven dried (55 °C) until constant weight and pulverized using a roller grinder^43^. Bulk density of each 15 cm depth segment was determined using standard methods^44^. Ground subsamples of each soil depth segment were analyzed for C and N using a LECO TruSpec CN Analyzer (LECO Corp., Saint Joseph, MI). Following study completion in November 2011, two soil cores from each treatment combination along with BS soil cores from each block were collected (using the above method^42^) immediately prior to plant destructive harvest. Soil C and N data for each substrate were analyzed individually for each species and also across all species for each 15 cm soil depth increment. Due to minimal N, sparse treatment effects and for sake of brevity, soil N concentrations are not reported.

On December 3, 2011, all plants were destructively harvested. Plant shoots were cut at 15.24 cm above the soil line. Roots were extracted by attaching a clamp to the stump, connecting the clamp to a hydraulic cylinder mounted on the front of a small tractor and raising the cylinder mount until the taproot and lateral roots were loosened from the soil^42^; additional visible roots were collected by hand. Following destructive harvest, shoots and roots were dried in a forced air oven (55˚C for 14 days) and dry weights (DW) were recorded. Plant shoot and root subsamples were ground (2 mm sieve) and analyzed for C and N using a LECO TruSpec CN Analyzer. All soil C and plant biomass data were analyzed using the mixed model procedures (Proc Mixed) in SAS (SAS^®^ Institute version 9.1, Cary, NC). Means were separated using Fisher’s Least Significance test and in all cases differences were considered significant at *P* < 0.05.

Soil respiration data (ACES) were first analyzed for system and power failures with obvious systematic errors being parsed from the data set^40^. A total of 76,648 soil CO_2_ efflux observations were taken over the course of the study with 95.6% being considered acceptable for use in analysis. Species and substrate main effects comparisons of soil CO_2_ efflux were made using the LSmeans statement in Proc Mixed; the LSmeans slice option was used to test simple effects of substrate within each species with multiple comparisons being made using the pdiff option (*P* < 0.05) (SAS). Species and substrate simple and main effects were determined by week (data not shown), month (data not shown), season, entire measurement period average and cumulatively over the course of the experiment. Due to system constraints, ACES chambers measuring BS efflux could not be placed at each block within each species. Consequently, to analyze BS data in comparison to each species and substrate combination, BS effluxes were assumed to not greatly differ from the native soil in which the plants were transplanted. Since the entire experimental area was relatively small (400 m^2^), remained fallow for several years before study initiation and was managed in an identical manner, the native soil profiles were likely similar in all plots. Supporting this contention, soil tests from previous years indicated little or no variability in soil characteristics within the experimental site (Marble C, 2009, unpubl. data).

Linear correlations were determined for the effects of soil temperature (5 cm depth) on soil CO_2_ efflux using the Proc Corr procedure in SAS. All ACES data were averaged for 1.0 °C intervals of soil temperature. Averaging based on 1.0 °C increments was done in order to reduce the influence of outliers on the response of CO_2_ efflux to temperature over the course of the study. A similar procedure was used to investigate the relationship between soil CO_2_ efflux and soil moisture^40^,^45^,^46^. Linear regression was then used on the averaged data to determine the relationship between soil CO_2_ efflux and soil temperature or soil moisture. All data were considered significant at *P* < 0.05.

## Results

Potting substrate had no effect on plant shoot or root DW in all three species (Table 1). Additionally, no differences in shoot or root C or N were observed. Growth data indicated all three species performed similarly regardless of potting substrate used in container production, similar to previous results reported by Marble *et al.*^38^.

**Figure 2:**
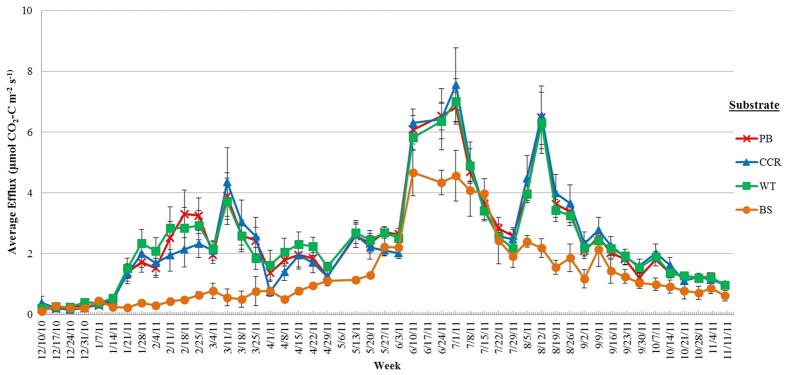
Main effect of substrate on weekly CO_2_-C efflux, averaged across all three species. Mean weekly averages and standard errors are shown. PB = pinebark; CCR = clean chip residual; WT = WholeTree; BS = bare soil (no species or substrate).

**Figure 3:**
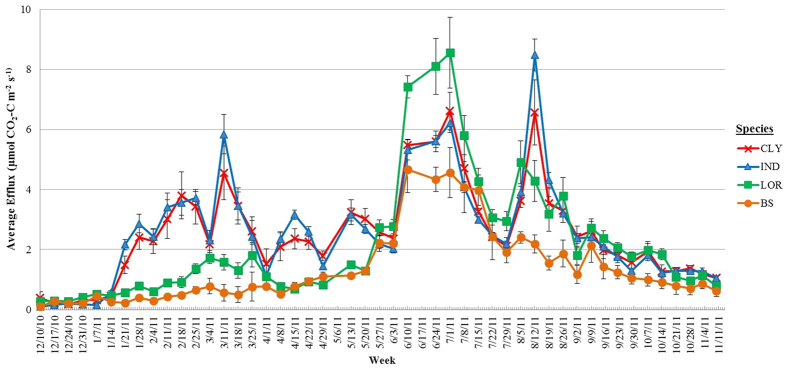
Main effect of species on weekly CO_2_-C efflux, averaged across all three substrates. Mean weekly averages and standard errors are shown. CLY = cleyera; IND = Indian hawthorn; LOR = loropetalum; BS = bare soil (no species or substrate).

Weekly substrate main effects showed no clear pattern among the three potting substrates, other than PB, CCR and WT having higher efflux than BS on most occasions (Fig. 2). Species main effects were significant for most weeks with the general trend showing loropetalum having either a lower (weeks 12 to 21; 24 to 30) or higher (weeks 32 to 40) efflux than either cleyera or Indian hawthorn which displayed similar efflux throughout most of the study (Fig. 3). Average soil CO_2_ efflux for each species (across all substrates) for the study duration showed that Indian hawthorn had a slightly higher efflux rate (2.66 μmol CO_2_-C m^−2^ s^−1^) than loropetalum (2.09 μmol CO_2_-C m^−2^ s^−1^; *P* = 0.0458), while cleyera (2.52 μmol CO_2_-C m^−2^ s^−1^) was similar to both species (Table 2). All species had higher efflux than BS (1.29 μmol CO_2_-C m^−2^ s^−1^; Table 2) and no additional species or substrate effects were significant. Seasonal averages for each substrate (across all species) showed that PB, CCR and WT had similar effluxes; all had higher efflux than BS with the exception of Fall 2011 when BS was similar (Table 3). While plots containing potting substrate had higher efflux than BS, no other substrate effects were significant. Across all substrates, species effects were significant in winter 2010 (*P* = 0.006) where loropetalum had lower efflux than cleyera (*P* = 0.0003) and Indian hawthorn (*P* = 0.0001); similar results were also observed in spring 2011. Overall (Table 2) and seasonal average efflux (Table 3) results were unexpected given that loropetalum had over 100% more root biomass than Indian hawthorn (Table 1) and would be expected to have had a higher autotrophic respiration rate. Simple main effects showed that WT plots in cleyera (*P* = 0.0027) and Indian hawthorn (*P* = 0.0028) and CCR plots in Indian hawthorn (*P* = 0.0382) had higher efflux when compared to WT or CCR plots in loropetalum. The reason for this difference is unknown, but was possibly a consequence of species effects on heterotrophic factors not captured and will likely require further investigation in future work. No species or media effects on cumulative efflux were observed (Table 4) other than plots with plants having higher efflux than BS.

Soil efflux has been shown to increase with temperature in most soil types^45^,^46^ and efflux in this study was generally higher during warmer spring and summer months (Table 3). There was a significant positive correlation between soil temperature (ST) and soil efflux [(efflux = 0.143 + (0.1097*ST)); R^2^ = 0.8336, *P*  <0.0001, data not shown]; this trend can also be observed in Figs 2 and 3. Soil moisture (SM) showed a weak negative linear correlation (R^2^ = 0.2421) with soil CO_2_ efflux; however, these data showed a better fit when a quadratic function was employed [(efflux = −3.3536 + (0.4262*SM) − (0.0077*SM^2^)); R^2^ = 0.6865, P <0.0001, data not shown].

Soil C content in 2009 at the 0–15 cm depth showed higher C in plots containing potting substrate as compared to BS (Table 5). While all substrates had similar C levels in Indian hawthorn, PB (8.8 kg C m^−2^) had higher C content than WT (5.7 kg C m^−2^) in cleyera and had the highest C of any substrate in loropetalum (12.6 kg C m^−2^). When soil C content was averaged across all species for each substrate, soil C was highest in PB (10.6 kg C m^−2^), followed by CCR (7.6 kg C m^−2^), WT (6.0 kg C m^−2^) and BS (3.0 kg C m^−2^). Following transplanting, the majority of substrate was contained within the top 15 cm depth, as shown by low C levels and sparse differences among treatments at lower depths (15–60 cm). At study conclusion in 2011, PB had higher C content than CCR or WT at the 0–15 cm depth in all three species and when averaged across species. A comparison of substrate mean C content at the 0–15 cm depth from 2009 to 2011 showed that C levels in PB had not declined significantly (10.6 to 10.1 kg C m^−2^; *P* = 0.4730); this was not the case for CCR (7.6 to 5.5 kg C m^−2^; *P* = 0.0017) or WT (6.0 to 4.3 kg C m^−2^; *P* = 0.0164). As in 2009, few differences were noted in 2011 for soil C at lower depths.

## Discussion

Previous reports have illustrated slower decomposition of bark when compared to wood. Allison and Murphy^47^ investigated wood and bark decomposition rates of several pine species, including loblolly pine (*Pinus taeda* L.) which was the species used for CCR and WT substrates in this study; results showed that ~16.9% of wood C was oxidized compared to 8.6% for bark. Bark generally decomposes slower than wood due to its high lignin content^48^,^49^,^50^. Clean chip residual and WT are composed of ~50 and 80% wood, respectively^34^,^35^, and would be expected to decompose faster than PB. An incubation study by Boyer *et al.*^51^ showed that CCR exhibited a high microbial respiration rate that was similar to PB; this may be attributable to the bark content (40%) in the CCR substrate. In the current study, PB and CCR had similar soil C levels only in the top 0–15 cm depth for cleyera and Indian hawthorn in 2009. By 2011, CCR soil C levels were similar to WT levels, but lower than PB. Lower C levels in CCR and WT plots were likely attributable to the high wood percentage in these substrates that had ample time to decompose by 2011 (Table 5). Additionally, when averaged across all species, CCR and WT soil C levels in the top depth decreased in 2011 compared to 2009, while PB levels remained similar. Results indicate that PB likely has greater longevity in the soil following transplanting than CCR or WT, thereby resulting in a greater long-term C gain.

Soil CO_2_ efflux generally increases with temperature^40^,^45^,^52^, as was observed in the current study. The slightly better fit seen in the study by Runion *et al.*^40^, which also reported ACES data, can be attributed to the fact that their study ran for only 90 days across one season and the current study ran for a full year. The longer duration captured the influence of seasonal variations in temperature on soil CO_2_ efflux where rates were generally higher in the warmer Spring and Summer months (Figs 2 and 3).

Soil moisture also influences soil CO_2_ efflux due to both physical (soil gas displacement) and biological (autotrophic and heterotrophic respiration) mechanisms^52^. Runion *et al.*^40^ also saw a good fit of soil moisture to CO_2_ efflux using a quadratic equation, as observed here, suggesting there is a moisture content at which CO_2_ efflux is maximized for a given soil type. As with soil temperature, the lower R^2^ seen in the present study (vs. Runion *et al.*^40^) reflects the seasonal effects of soil moisture on CO_2_ efflux. For example, the highest soil moisture readings occurred during the cooler Fall and Winter months which generally had the lowest soil CO_2_ efflux rates, while higher rates in the Spring and Summer occurred across a wider soil moisture range.

Net C gain was estimated by extrapolating cumulative soil efflux data over the three years following landscape transplanting. Estimated total cumulative soil efflux (kg C m^−2^) for each species over the course of the study were 2.7 (PB), 2.6 (CCR) and 3.1 (WT) for cleyera; 3.0 (PB), 2.8 (CCR) and 2.8 (WT) for Indian hawthorn; and 2.4 (PB), 2.6 (CCR) and 2.3 (WT) for loropetalum. Estimated total cumulative soil efflux from BS was 1.5 kg C m^−2^. Subtracting extrapolated cumulative soil C efflux from 2011 soil C content (at top depth only) gave an estimated net C gain (kg C m^−2^) of 4.6 (PB), 1.7 (CCR) and 1.0 (WT) for cleyera; 6.5 (PB), 3.1 (CCR) and 1.3 (WT) for Indian hawthorn; and 8.8 (PB), 3.5 (CCR) and 2.6 (WT) for loropetalum; BS was estimated to be C neutral. When the above calculations (in kg m^−2^ for the top soil depth) were converted to reflect the area of the container used during initial production (0.025 m^2^), estimated average C gains for PB, CCR and WT were 165.8, 69.0 and 48.0 g, respectively. Note that these efflux estimates (and consequently net C estimates) were taken from only one year of data and do not account for changes in plant respiration rates which can be influenced by plant age, size and environmental factors^53^. Further, since substrate had little effect on efflux rate, it is possible that more rapid substrate decomposition occurring in the first few months to 1 year after planting was not captured in this study. However, since all substrates had similar initial C levels, it is likely that the final C levels reflect that PB decomposed slowest, followed by CCR and WT.

Additional C gains would be realized by considering C accumulation in growing plants which was mostly dependent on plant size since shoot and root C was generally similar among species (Table 1). It should also be noted that efflux data from this study do not include autotrophic respiration from the plants shoots which would slightly affect net C estimates.

Data suggest that planting container-grown woody ornamental shrubs in homeowner landscapes significantly contributes to C sequestration, with total net C gain being influenced by potting substrate. Biomass production is known to be a major source of C accumulation; our results also showed that potting substrate should be considered in the overall C sequestration potential of homeowner landscapes when plants were originally container-grown. As most woody shrubs require little or no intensive maintenance, this C gain is likely higher than gains reported for ornamental turf areas (140 g C m^−2^ yr^−1^) that require higher chemical and mechanical inputs^54^. Determining the overall impact of the landscape industry on C sequestration should include other initial production factors such as fertilizer, irrigation, farm energy use and equipment fuel consumption in future investigations. However, previous research has shown that container-grown ornamentals typically act as a net C sink while still in production^30^,^55^ and continue to provide this benefit after transplanting^29^. Results from this research indicate that planting container-grown ornamental shrubs allows homeowners a means of directly contributing to C sequestration while increasing property values and aesthetics. If future C emissions are “capped” or taxed as speculated^56^,^57^,^58^, the ability to show environmental benefits of landscaping private and public properties will become vital. These results also illustrate the importance of potting substrates as a vehicle for C sequestration that has been ignored in previous research. As the ability of plants to sequester C is well understood, future work should focus on the C storage potential of soilless media in landscape horticulture.

## Additional Information

**How to cite this article**: Marble, S. C. *et al.* Species and Media Effects on Soil Carbon Dynamics in the Landscape. *Sci. Rep.* **6**, 25210; doi: 10.1038/srep25210 (2016).

## Figures and Tables

**Table 1:** Biomass, carbon and nitrogen concentration of plant shoots and roots^z^.

Media^y^	Shoots			Roots		
	Dry wt. (g)	Carbon %	Nitrogen %	Dry wt. (g)	Carbon %	Nitrogen %
	Cleyera					
PB	809.6 a	47.1 a	1.2 a	498.1 a	48.1 a	0.5 a
CCR	927.3 a	47.1 a	1.2 a	524.5 a	48.0 a	0.6 a
WT	773.7 a	47.2 a	1.1 a	495.5 a	47.7 a	0.5 a
	Indian Hawthorn					
PB	1775.9 a	47.3 a	1.2 a	325.0 a	48.1 a	0.7 a
CCR	1900.9 a	47.4 a	1.1 a	341.5 a	48.1 a	0.6 a
WT	1361.1 a	47.3 a	1.1 a	254.1 a	48.2 a	0.6 a
	Loropetalum					
PB	2653.2 a	44.7 a	1.0 a	735.2 a	45.8 a	0.4 a
CCR	3036.6 a	44.7 a	1.0 a	885.3 a	45.9 a	0.4 a
WT	2752.9 a	44.7 a	1.0 a	772.5 a	45.8 a	0.4 a

^z^Shoots show the carbon and nitrogen concentration of all above ground plant material (leaves, stems,and branches) (n = 6). Roots show the carbon and nitrogen content of belowground plant material (roots only) (n = 6).

^y^PB = pine bark; CCR = clean chip residual; WT = WholeTree.

^x^Means separated using Fisher’s Least Significance Difference Test (*P* < 0.05). Means within a column under each subheading followed by the same letter are not significantly different.

**Table 2:** Effects of species and potting substrate on average soil CO_2_ efflux, December 2010–November 2011.

Species^y^	Average	Substrate^x^	Average
Species effects on soil CO_2_ efflux^z^ across all media		Substrate effects on soil CO_2_ efflux across all species	
CLY	2.52 a^w^	PB	2.42 a
IND	2.66 a	CCR	2.42 a
LOR	2.10 a	WT	2.43 a
BS	1.29 b	BS	1.29 b
Species effects on soil CO_2_ efflux in PB		Substrate effects on soil CO_2_ efflux in CLY	
CLY	2.47 a	PB	2.47 a
IND	2.75 a	CCR	2.36 a
LOR	2.03 a	WT	2.75 a
BS	1.29 b	BS	1.29 b
Species effects on soil CO_2_ efflux in CCR		Substrate effects on soil CO_2_ efflux in IND	
CLY	2.36 a	PB	2.75 a
IND	2.60 a	CCR	2.60 a
LOR	2.30 a	WT	2.62 a
BS	1.29 b	BS	1.29 b
Species effects on soil CO_2_ efflux in WT		Substrate effects on soil CO_2_ efflux in LOR	
CLY	2.75 a	PB	2.03 a
IND	2.62 a	CCR	2.30 a
LOR	1.93 a	WT	1.93 a
BS	1.29 b	BS	1.29 b

^z^Soil CO_2_ efflux is presented in μmol (CO_2_-C m^−2 ^s^−1^).

^y^CLY = cleyera, IND = Indian hawthorn, LOR = loropetalum, BS = bare soil (no substrate or species) (n = 6).

^x^PB = pinebark, CCR = clean chip residual, WT = wholetree (n = 6).

^w^Differences in LSmeans within a column under each subheading with the same letter are not significantly different (*P* < 0.05).

**Table 3:** Average seasonal^z^ soil CO_2_ efflux among all species as affected by potting substrate.

Media^y^	Fall 2010	Winter 2010	Spring 2011	Summer 2011	Fall 2011
	Soil CO_2_-C efflux (μmol** **CO_2_-C** **m^−2^ s^−1^) across all species				
PB	1.22 a^x^	0.93 a	2.52 a	4.22 a	2.16 a
CCR	1.15 a	0.94 a	2.36 a	4.28 a	2.35 a
WT	1.21 a	1.03 a	2.51 a	4.12 a	2.18 a
BS	0.71 b	0.53 b	0.70 b	2.80 b	1.40 a
	Soil CO_2_-C efflux (μmol** **CO_2_-C** **m^−2^ s^−1^) in cleyera				
PB	1.37 a	0.90 a	2.86 a	4.02 a	2.24 a
CCR	1.10 a	1.05 a	2.84 a	3.65 a	2.04 a
WT	1.27 a	1.23 a	3.10 a	4.31 a	2.34 a
BS	0.71 b	0.53 b	0.70 b	2.80 a	1.40 a
	Soil CO_2_-C efflux (μmol** **CO_2_-C** **m^−2^** **s^−1^) in Indian hawthorn				
PB	1.37 a	1.13 a	3.22 a	4.32 a	2.26 a
CCR	1.14 a	1.07 a	3.37 a	3.99 a	1.93 a
WT	1.27 a	1.23 a	3.16 a	3.95 a	2.15 a
BS	0.71 b	0.53 b	0.70 b	2.80 a	1.40 a
	Soil CO_2_-C efflux (μmol** **CO_2_-C** **m^−2^** **s^−1^) in loropetalum				
PB	1.18 a	0.78 a	1.48 a	4.31 ab	1.97 ab
CCR	0.95 ab	0.69 a	0.87 a	5.19 a	3.08 a
WT	0.97 ab	0.64 a	1.27 a	4.10 ab	2.06 ab
BS	0.71 b	0.53 a	0.70 b	2.80 b	1.40 b

^z^Fall 2010 = 12/5/10 through 12/21/10; Winter 2010 = 12/22/10 through 3/19/11; Spring 2011 = 3/20/11 through 6/20/11; Summer 2011 = 6/21/11 through 9/22/11; Fall 2011 = 9/23/11 through 11/14/11.

^y^PB = pinebark, CCR = clean chip residual, WT = wholetree, BS = bare soil (no substrate) (n = 6).

^x^Differences in LSmeans within a column under each subheading with the same letter are not significantly different (*P* < 0.05).

**Table 4:** Substrate effects on cumulative soil CO_2_ efflux.

Substrate^y^	Cleyera	IndianHawthorn	Loropetalum
	Cumulative^z^ Efflux (g CO_2_-C m^−2^)		
PB	898.5 a^x^	994.2 a	788.5 a
CCR	856.4 a	922.4 a	868.4 a
WT	1027.5 a	933.6 a	759.6 a
BS	508.6 b	508.6 b	508.6 b

^z^Cumulative efflux was calculated using the trapezoid rule (December 5, 2010 through

November 14, 2011).

^y^PB = pinebark, CCR = clean chip residual, WT = wholetree, BS = bare soil (no substrate

or species).

^x^Differences in LSmeans within a column under each subheading with the same letter are not significantly different (*P* < 0.05) (n = 6).

**Table 5:** Effects of substrate on soil carbon content, 2009 and 2011.

	Soil Depth											
	0–15 cm			15–30 cm			30–45 cm			45–60 cm		
	Year		*P*^z^	Year		*P*	Year		*P*	Year		*P*
	2009	2011		2009	2011		2009	2011		2009	2011	
Substrate^y^	Soil C content^x^ (kg m^−2^) across all species											
PB	10.6 a^w^	10.1 a	0.4730	1.1 a	1.2 a	0.6224	0.6 a	0.6 a	0.8802	0.4 a	0.5 a	0.5481
CCR	7.6 b	5.5 b	0.0017	1.0 ab	1.3 a	0.2941	0.6 a	0.7 a	0.3921	0.4 a	0.6 a	0.2974
WT	6.0 c	4.3 bc	0.0164	0.8 b	0.9 a	0.5823	0.5 a	0.6 a	0.4361	0.7 a	0.4 a	0.1481
BS	3.0 d	2.8 c	0.7846	1.2 a	1.0 a	0.7381	0.5 a	0.6 a	0.6598	0.4 a	0.4 a	0.9754
	Soil C content (kg m^−2^) in cleyera											
PB	8.8 a	7.3 a	0.2023	1.2 a	1.2 a	0.9291	0.9 a	0.4 a	0.0823	0.5 a	0.3 a	0.6079
CCR	7.3 ab	4.3 b	0.1650	1.1 a	1.5 a	0.2804	0.6 a	0.7 a	0.5499	0.5 a	0.9 a	0.1769
WT	5.7 b	4.1 b	0.1809	0.9 a	0.8 a	0.9113	0.5 a	0.6 a	0.6429	1.5 a	0.3 a	0.0009
BS	2.5 c	3.2 b	0.5880	0.9 a	1.1 a	0.7467	0.4 a	0.6 a	0.5716	0.3 a	0.4 a	0.8549
	Soil C content (kg m^−2^) in Indian hawthorn											
PB	10.5 a	9.5 a	0.4336	1.1 b	0.7 a	0.3182	0.5 a	0.8 a	0.3017	0.3 a	0.8 a	0.1488
CCR	7.4 a	5.9 b	0.1772	1.0 b	1.2 a	0.7613	0.5 a	0.9 a	0.1585	0.4 a	0.6 a	0.5755
WT	7.3 a	4.1 bc	0.0060	0.8 b	0.8 a	0.8828	0.5 a	0.6 a	0.6756	0.4 a	0.5 a	0.6890
BS	3.4 b	2.3 c	0.3944	1.6 a	0.8 a	0.0598	0.7 a	0.6 a	0.7513	0.5 a	0.5 a	0.8612
	Soil C content (kg m^−2^) in loropetalum											
PB	12.6 a	13.3 a	0.4217	1.1 a	1.8 a	0.0894	0.5 a	0.6 a	0.6090	0.3 a	0.4 a	0.8816
CCR	8.2 b	6.1 b	0.0752	0.9 a	1.1 a	0.6714	0.6 a	0.4 b	0.5906	0.4 a	0.4 a	0.9132
WT	5.1 c	4.9 b	0.8953	0.7 a	1.2 a	0.2457	0.4 a	0.5 ab	0.6400	0.3 a	0.5 a	0.5759
BS	3.2 c	3.0 c	0.8329	0.9 a	1.3 a	0.3731	0.5 a	0.6 a	0.6325	0.3 a	0.3 a	0.9430

^z^Associated *P* values show comparison of each substrate soil C level in 2009 and 2011 at each depth sampled.

^y^PB = pinebark, CCR = clean chip residual, WT = wholetree, BS = bare soil (no substrate) (n = 6).

^x^Soil carbon content shows levels of C (kg) containted at each soil depth over 1 m^2^.

^w^Means separated using Fisher’s Least Significance Difference Test (*P* < 0.05). Means within a column under each subheading followed by the same letter are not significantly different.
